# 

**Published:** 1916-04

**Authors:** 


### CONTENTS, APRIL, 1916—VOLUME XXXI, NO. 10.

### ORIGINAL ARTICLES.

Vincent's Angina. By Dr. Ray Karchmer Daily, Houston, Texas.. 393

Difficulties in Breast Feeding. By Dr. Mary Cleveland Harper, San Antonio ........ 397

"Clean Milk." By Dr. Ethel Lyon Heard, Galveston, Texas...... 406

An Unusual Case of Renal Tuberculosis. By Dr. Violet H. Keiller, Galveston, Texas ........ 409

Abnormal Delinquency. By Dr. Minnie L. Maffett, Dallas, Texas.. 411

The Examination of Blood Smear as a Diagnostic Asset. By Dr. Martha A. Wood, Houston, Texas........ 420

### EDITORIALS.

To Subscribers ........ 425

Our Forthcoming Series of Articles........ 425

This Number of the JOURNAL........ 426

This Is Your Journal........ 427

Dr. White's Articles........ 428

Items of General Interest........ 428

Personals ........ 431

Changes of Location........ 434

Deaths ........ 434

Book Reviews ........ 436

Helpful Hints for the Busy Doctor........ 438

